# The onset of motor learning impairments in Parkinson’s disease: a computational investigation

**DOI:** 10.1186/s40708-023-00215-6

**Published:** 2024-01-29

**Authors:** Ilaria Gigi, Rosa Senatore, Angelo Marcelli

**Affiliations:** 1 Institute of Cognitive Sciences and Technologies (ISTC), National Research Council of Italy (CNR), Via Beato Pellegrino 28, Padova, 35137 Veneto Italy; 2 Natural Intelligent Technologies Ltd, Piazza Vittorio Emanuele 10, Fisciano, 84084 Campania Italy; 3 Department of Information Engineering, Electrical Engineering, and Applied Mathematics (DIEM), University of Salerno, Via Giovanni Paolo II 132, Fisciano, 84084 Campania Italy

**Keywords:** Basal ganglia, Motor learning, Neural network model, Parkinson’s disease, Reinforcement learning

## Abstract

The basal ganglia (BG) is part of a basic feedback circuit regulating cortical function, such as voluntary movements control, via their influence on thalamocortical projections. BG disorders, namely Parkinson’s disease (PD), characterized by the loss of neurons in the substantia nigra, involve the progressive loss of motor functions. At the present, PD is incurable. Converging evidences suggest the onset of PD-specific pathology prior to the appearance of classical motor signs. This latent phase of neurodegeneration in PD is of particular relevance in developing more effective therapies by intervening at the earliest stages of the disease. Therefore, a key challenge in PD research is to identify and validate markers for the preclinical and prodromal stages of the illness. We propose a mechanistic neurocomputational model of the BG at a mesoscopic scale to investigate the behavior of the simulated neural system after several degrees of lesion of the substantia nigra, with the aim of possibly evaluating which is the smallest lesion compromising motor learning. In other words, we developed a working framework for the analysis of theoretical early-stage PD. While simulations in healthy conditions confirm the key role of dopamine in learning, in pathological conditions the network predicts that there may exist abnormalities of the motor learning process, for physiological alterations in the BG, that do not yet involve the presence of symptoms typical of the clinical diagnosis.

## Introduction

Parkinson’s disease (PD) is a neurodegenerative disorder inducing several motor symptoms (tremor, rigidity, akinesia) and cognitive deficits (regarding procedural learning and decision making).

PD is characterized by the death of dopaminergic neurons projecting to the Basal Ganglia (BG), a group of nuclei part of several anatomical and functional loops, involving the cerebral cortex and the thalamus.

The BG is involved in, among others, voluntary movements control, procedural learning, decision making, cognition, and emotion. Its primary function is that of controlling and regulating the activities of the motor and premotor cortical areas for executing smooth movements. Various authors [1–6] agree on the role of BG in selectively facilitating the execution of a single elementary motor command while suppressing all competing others. Current theories of motor learning claim that early learning is controlled by dopamine-modulated plasticity in the BG, which trains parallel cortical pathways through unsupervised plasticity at corticostriatal synapses [7, 8].

After more than a century of work on the motor functions of BG, in the last decades, researchers have focused on the function in learning habits and acquiring motor skills. Much evidence [7, 9–11] has been collected on the role of BG in refining action selection according to an optimization process and shaping skills as a modulator of motor repertoires; this learning mechanism supported by striatal circuitry generalizes to other domains, including cognitive skills.

People affected by PD show a degradation of motor learning performance [12–14]. As a consequence, many researchers are investigating the features of motor degradation in PD, with the aim of determining specific therapies and technologies to counteract, at least partially, the symptoms of the disease. Manipulations of dopamine levels in the striatum in animal models have been shown to affect the activation of behavior and the development of skilled motor responses [15]. It has also been shown that the BG participates in cognitive decision-making, similarly to the context of motor control, since the loops linking the BG with frontal systems are very similar, and because of cognitive deficits observed in PD patients [16, 17], so naturally extending the role of action selection to high-level cognitive decisions.

Given its key role in the motor and cognitive context, the functional role of the BG and the dopaminergic system represents an issue of particular interest. Furthermore, as no definitive biomarker of PD has been defined, an urgent need exists to develop early diagnostic biomarkers for two reasons: (1) to intervene at the onset of the disease and (2) to monitor the progress of therapeutic interventions that may slow or stop the course of the disease.

When system-level interactions of multiple brain regions are involved, or when these structures are subcortically located, computational investigations provide a valuable complement to experimental brain research. Exploration of the neural interactions through a computational model could gain some insights about the functional dynamics of information processing within the simulated brain areas, in normal as well as in diseased brains, providing also some guidelines for developing more efficacious therapies for diseases in which these areas are involved. Indeed, modeling approaches could be applied to develop testable “computational biomarkers” to support diagnostic, prognostic, or treatment efforts, particularly, on the path to “digital twin” approaches in brain medicine.

In this light, we have developed a biologically inspired mechanistic model of the cortico-BG-thalamo-cortical loop, based on neuroanatomical studies, experimental findings, and computational studies, that mimics information processing in the BG and simulates the performance of motor tasks at some level of abstraction.

## The Basal Ganglia and the dopaminergic system: anatomical and functional aspects

The BG is a group of subcortical nuclei, situated at the base of the forebrain and top of the midbrain. These nuclei are strongly interconnected with several brain areas and associated with a variety of functions, including control of voluntary motor movements, procedural and habit learning, cognition, and emotion.

### The Basal Ganglia

In the context of motor control, the BG facilitates the execution of appropriate actions and suppresses the inappropriate ones; in other words, the BG modulates motor responses rather than encoding any detail of them, releasing the brake on the motor command winning the competition (i.e., getting executed). Action selection by the BG, i.e. the choice of the motor responses, is implemented by a sequence of parallel loops of connectivity. Each region of the cortex has a corresponding BG circuit for gating proper initiation of a specific movement. The motor circuit has been examined in experimental studies and has been implicated in a wide range of motor behaviors, particularly in the high-level aspects of movement, such as the preparation of movement or the control of kinematic parameters. The BG integrates information from different brain structures into an appropriate motor response, e.g., in the reaction to sensory stimuli or also in everyday movement and the fine control of all our movements, such as walking, writing, and so on.

Therefore, the BG modulates the efficacy of responses being selected in the cortex. This suggests that the BG does not initiate motor responses, but rather facilitates or gates responses that are being considered in the premotor cortex. Looking at it another way, the BG modulates elementary actions, which are then pieced together by the nervous system to generate a complex motor behavior, that results from the concatenation of elementary movements. In this complex system, the BG acts by enhancing the most appropriate command at any given portion of the sequence of actions.

**Fig. 1:**
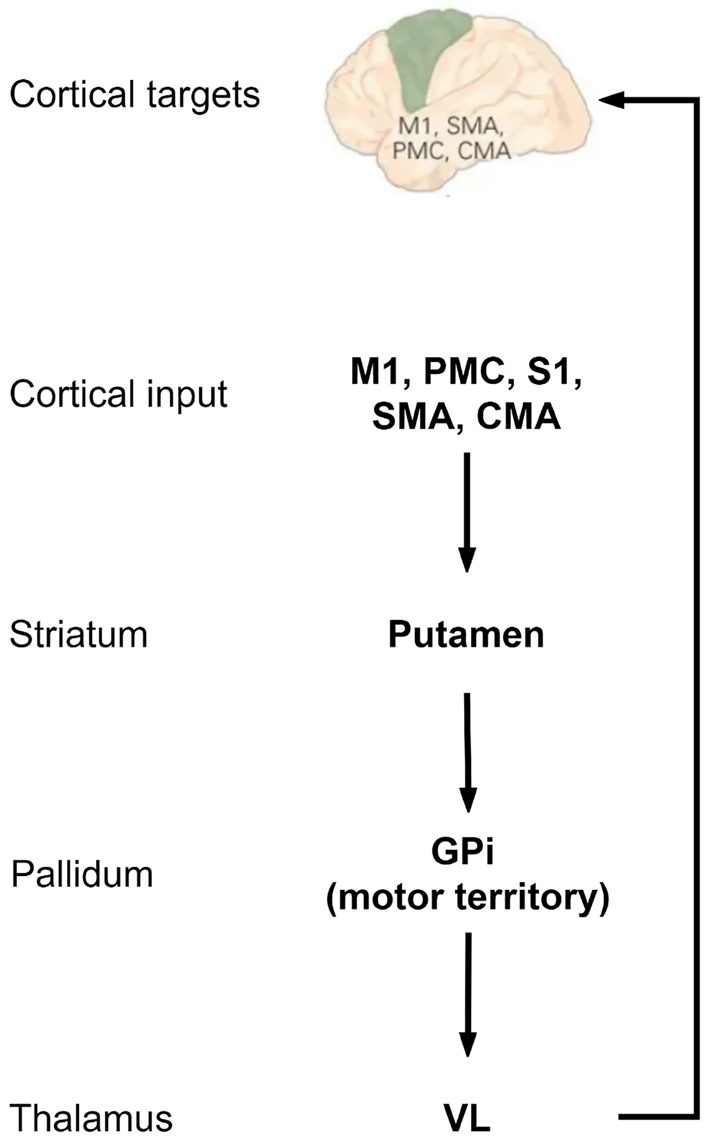
Basal Ganglia functional loop: motor circuit for body movement. Primary motor cortex (M1), premotor cortex (PMC), primary somatosensory cortex (S1), supplementary motor area (SMA), cingulate motor area (CMA), internal globus pallidus (GPi), ventrolateral (VL) thalamus

The nuclei composing the BG receive (send) connections from (to) several brain areas. In the motor loop, the associated striatum (putamen) receives input primarily from the motor cortex, namely, the supplementary motor area (SMA), cingulate motor area, premotor cortex (PMC), and primary motor (M1), and also from parietal cortex, mostly primary somatosensory cortex (S1). These input signals, coming from a large region of the frontal–parietal areas, properly contextualize motor actions. A high-level diagram of the motor loop is reported in Fig. 1.

The inputs converging into the BG are processed and reissued, through the thalamus, to the cortex. The thalamus projects principally to frontal lobe areas (prefrontal, premotor, and supplementary motor areas) which are concerned with motor planning. From the motor cortex, ultimately the BG affects the planning and execution of the movement by synapsing with the neurons of the corticospinal and corticobulbar tracts in the brainstem and spinal cord [18, 19].

**Fig. 2:**
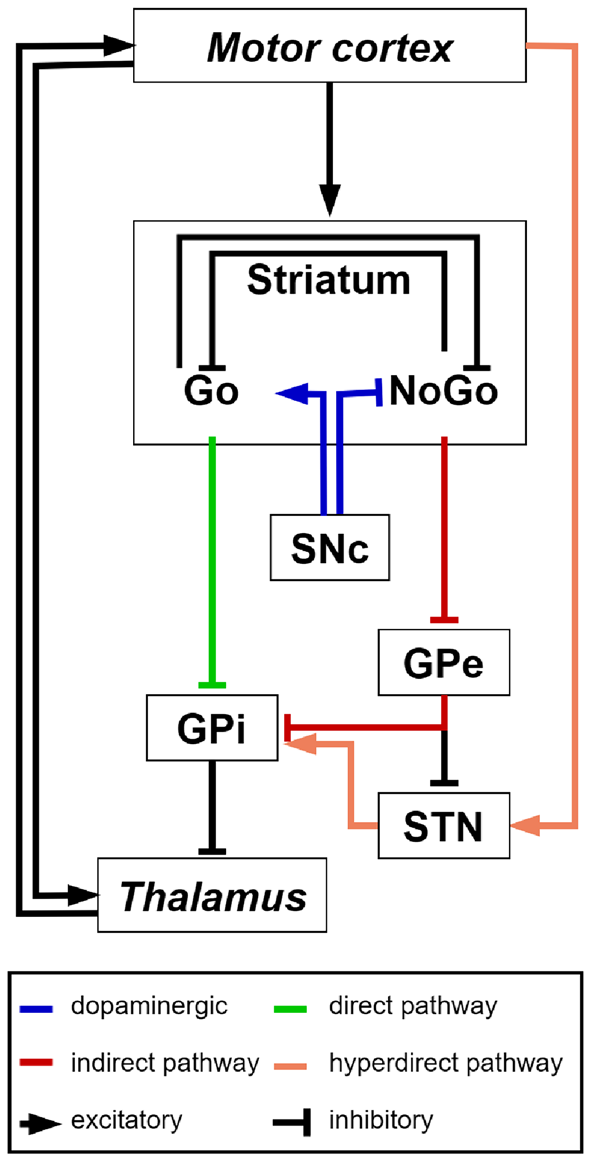
Topology connection diagram of the model. The corticostriatal–thalamocortical loop, including the direct, indirect, and hyperdirect pathways of the BG, is represented. The direct pathway-projecting Go neurons in the striatum project directly to the GPi, having the effect of disinhibiting the thalamus and executing a motor response represented in the cortex. The indirect pathway-projecting NoGo neurons have the opposite effect and suppress actions from getting executed. Dopamine from the Substantia Nigra pars compacta (SNc) projects to the striatum, exerting excitation of Go cells via D1 receptors, and inhibition of NoGo cells via D2 receptors. The Subthalamic nucleus (STN) receives excitatory projections from the cortex in the hyperdirect pathway and excites GPi; the external Globus Pallidum (GPe) provides an inhibitory effect on STN activity. Areas not belonging to BG are marked in italic

The BG system involves the following subregions: striatum, subthalamic nucleus (STN), substantia nigra pars compacta (SNc), the internal globus pallidus (GPi) and the external globus pallidus (GPe). These nuclei are interconnected by different pathways (see Fig. 2).

The input structure of the BG is the striatum (putamen) and receives inputs from two main sources: cortical areas (including the motor cortex), and the SNc (through dopaminergic projections).

Two main projection pathways go from the striatum to the output segment of BG, the GPi, up to the thalamus and back to the cortex, which have opposing effects on the excitation/inhibition of the thalamus (i.e., execution/suppression of the action). These pathways are named direct and indirect, respectively. The direct pathway facilitates the execution of responses, whereas the indirect pathway suppresses them.

Neurons from the direct pathway project from the striatum through an inhibitory connection to GPi, which is tonically active and inhibiting the thalamus. Thus, the excitation of the direct pathway results in the inhibition of the activity of GPi, which in turn ceases to inhibit the thalamus; the thalamus is enabled to get excited from other excitatory projections and thalamocortical projections propagate the excitation to the cortex, enhancing the activity of the motor response represented in the motor cortex, so that it can be executed (i.e., the brake is released).

Neurons in the indirect pathway inhibit the GPe, tonically active and inhibiting the GPi. Therefore, the excitation of the indirect pathway releases the tonic inhibition of GPe onto GPi, which in turn is more active and then further inhibits the thalamus, suppressing actions from getting executed, thereby having an opposite effect.

The output structure of the BG, the GPi, acts like the volume dial on a radio, because its output determines whether a movement will be weak or strong. This mechanism accounts for the interpretation, from a functional point of view, of direct pathway neurons sending a Go signal to execute a response, and indirect pathway neurons a NoGo signal to suppress all the others.

It is still controversial whether the direct and indirect pathways compete with each other, or are functionally independent. That is, ultimately only one of Go or NoGo pathways activity predominates in the excitation/inhibition of the GPi/thalamus, thus amplifying or decreasing the force of movement. As in other studies [2, 5, 20], here we adopt the distinction between two sub-populations of neurons in the striatum, based on differences in biochemistry and efferent projections.

Through the interaction of the direct and indirect pathway, the BG serves as a gate on the thalamocortical circuit, in that it modulates the execution or inhibition of a motor command by acting on the thalamus.

Another important BG pathway, involving the activity of the STN, is the hyperdirect pathway, so called because cortical projections toward the STN directly excite GPi, bypassing the striatum. The cortico-STN pathway has a substantial effect on modulating the time of response execution. The activation of STN enhances GPi activity and its inhibition of the thalamus, therefore reducing the probability of facilitating a specific response. As a consequence, the hyperdirect pathway represents a non-specific (with respect to response) excitatory process to cancel (all) inappropriate actions. The STN may be essential to allow all information required to make decisions to be integrated before facilitating one, and thereby prevents premature responding [4, 21–24].

### Learning function of the BG: the role of dopamine

Dopamine (DA) plays a crucial role in a variety of processes, including reinforcement learning, motivation, and working memory. Compelling evidence has shown that DA is the central player in the induction of plasticity at corticostriatal synapses on striatal neurons, in concert with other neurotransmitters [25, 26].

DA firing patterns fluctuate between two different modes: phasic and tonic. The phasic signal is fast-timescale and spans milliseconds, whereas the tonic signal is slow-timescale and can span minutes or hours. Phasic changes in DA play a key role in synaptic plasticity and reinforcement learning, as are thought to occur during error feedback (e.g., [2, 5, 20, 27]), causing the two sub-populations of striatal neurons (direct and indirect) to independently learn positive and negative reinforcement values of responses. In particular, phasic increases in DA, due to positive feedback, result in increased activity in the direct pathway and suppression of the indirect pathway. On the contrary, phasic dips in DA, due to negative feedback, have the opposite effect, releasing the indirect pathway from suppression.

Therefore, the phasic changes in DA are critical for modulating Go/NoGo representations in the BG and ultimately facilitate or suppress the execution of motor commands. In other words, DA phasic levels correspond to a non-specific reward-mediated trial-by-trial training signal, as it strengthens the synapses active on correct trials and weakens the synapses active on incorrect trials.

Striatal Go/NoGo associations are learned through phasic changes in simulated DA firing during positive and negative feedback, resulting in the modulation of synaptic plasticity and supporting learning. Bursts of DA lead the BG to learn to facilitate rewarding behaviors and sharpen representations in the Go pathway. Dips of DA allow the BG to learn NoGo representations to the incorrect responses, and suppress disadvantageous behaviors.

Current theories of motor learning pose that early learning is controlled in the BG by DA-modulated plasticity, which trains parallel cortical pathways through unsupervised plasticity as a motor task becomes well-learned. In human trial-and-error learning tasks, phasic bursts and dips of DA have been proved to occur during positive and negative feedback, respectively, and these changes in extracellular levels of DA during feedback are thought to be critical for learning because they modify synaptic plasticity; in other words, DA acts as a “teaching signal”, leading to the learning of rewarding behaviors and discouraging unrewarding ones [20, 28]. It has been widely agreed that learning impairment in PD is linked to damaged DAergic neurons in the BG and DA deficiency [25, 26, 29].

The core features of the functional role of the BG nuclei in motor learning are

- The cortex generates multiple competing candidate actions for a given sensory context, which are kept inhibited by tonic activity in the output structures of BG. The striatum can release the inhibition on one of these channels, such that the most rewarding one is chosen to be activated.
- Competitive dynamics between striatal cells in the direct and indirect pathways of the BG facilitate or suppress a given response in the cortex. The cells that detect conditions to facilitate a response provide a Go signal, whereas those that suppress responses provide an NoGo signal.
- DA has a key role in modulating both activity and plasticity in the BG. Phasic changes (bursts and dips) in DA during error feedback are critical for learning stimulus–response associations and for allowing Go/NoGo representations to facilitate or suppress the execution of a command.
- Less DA (as in the PD state) leads to less contrast enhancement and impairs the ability to resolve Go/NoGo association differences needed for discriminating between different responses.
- Hebbian learning enhances associated cortical representations in a learning process slower than the reinforcement-based one.
- The STN provides a global NoGo signal that suppresses all responses. By this account, cortico-subthalamic-pallidal pathways modulate the dynamics of action selection by regulating the threshold for executing a response.

## Materials and methods

We propose a biophysically detailed mechanistic model at a mesoscopic scale, which incorporates

- The competing processes of the direct and indirect pathways, that allow to gate (Go) the correct response and suppress (NoGo) the competing responses.
- The SNc, so that the role of DA can be implemented and manipulated, with simulated D1 and D2 receptors in the striatum. In particular, the model includes DA bursts and dips (increase of SNc unit firing for correct responses and decrease for incorrect responses) and the corresponding DA-based learning mechanism (based on a reinforcement learning paradigm inspired by the learning algorithm of Leabra [30]).
- The STN, which allows us to explore its contribution to providing a global modulatory signal on the facilitation and suppression of all responses.
- The STN dynamic effects, as the response selection process evolves, both within the trial and with the advance of learning.
- The M1 and the mechanisms of cortical synaptic plasticity.
- The procedural motor learning hypothesis, allowing the BG to initially learn which response to gate via phasic changes in DA, and then this learning transfers to the cortex. In other words, BG and DA critically mediate the acquisition of behavior, but they play a diminishing role in executing well-learned behaviors.

The implementation of the model includes four competing responses but can be extended proportionally to include more alternatives.

In the following we describe the computational features of our BG model interconnected with the other cortical (S1, PMC, and M1) and subcortical (thalamus) areas, including the key learning mechanisms, connectivity, and general simulation methods. The model, shown in Fig. 2, is derived from validated models of decision-making [3, 20, 31], and associates a motor command to a particular sensory state through a learning process; its neural elements interact to yield this emergent behavior.

### General methods

The neural network is implemented within NEST (NEural Simulation Tool) framework, using an implementation of a basic point-neuron conductance model, the conductance-based leaky integrate-and-fire (LIF) neuron model, which provides a standard biophysically grounded model of individual neuron dynamics.

The adoption of NEST enables an in-depth analysis, more extensively than other simulators, as it allows to setting and measuring of the firing pattern of a population of neurons and examining in detail the behavior of both the externally manipulated neurons and the intermediate ones in the network in terms of their neuronal activity (i.e., firing rate, synapse strength, or timing delay). Thus, it becomes possible to simulate neuronal lesions to various degrees, acting on the firing rate of neurons rather than simulating only the presence/absence of a neuron.

There are simulated excitatory and inhibitory synaptic input channels. Synaptic connection weights have been trained using a reinforcement learning algorithm.

**Fig. 3:**
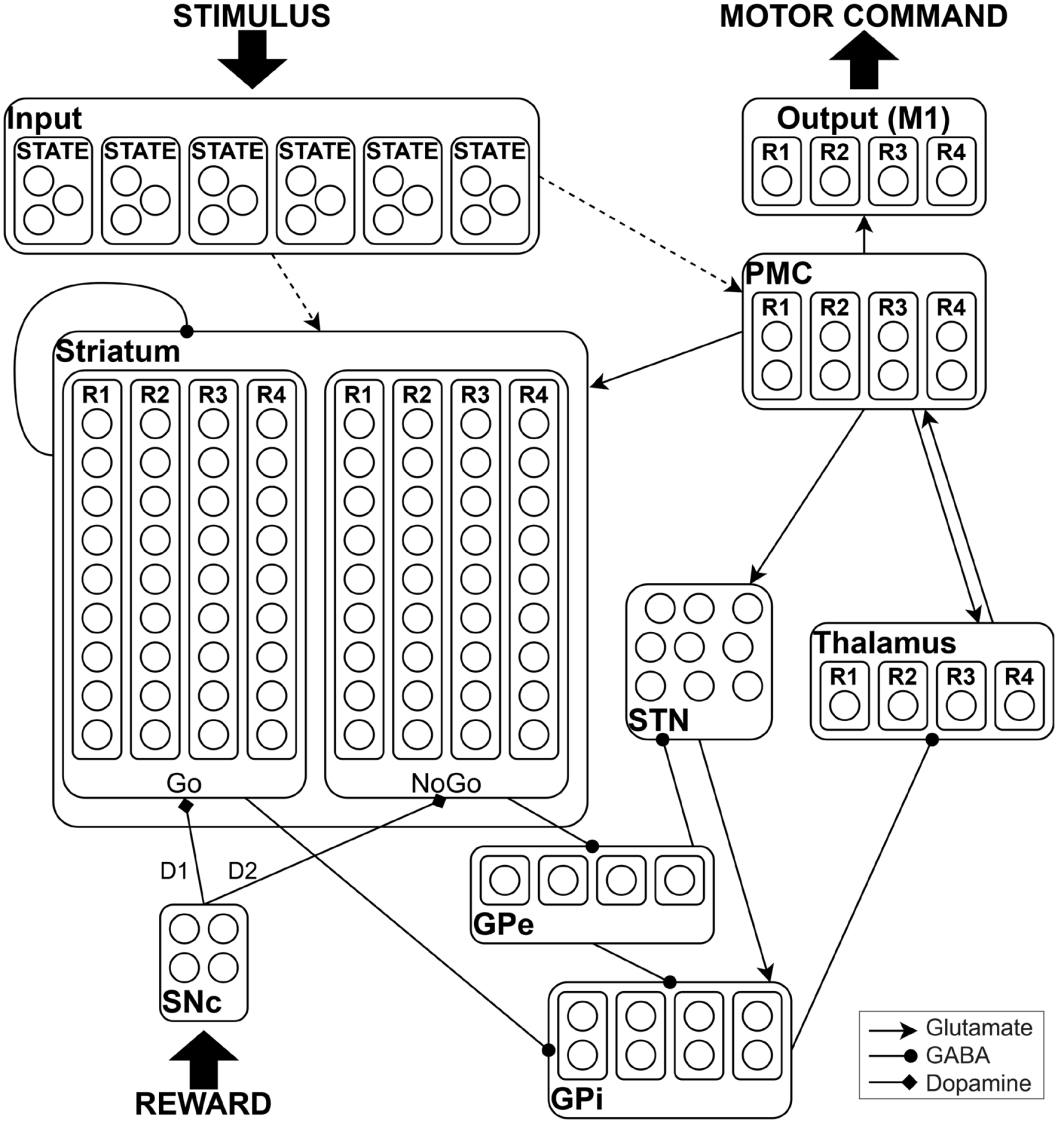
Network structure. Different brain areas are represented in separate layers. Internally, neurons are optionally grouped in columns. Dashed lines indicate plastic connections. The line end represents the influence of a neurotransmitter on the downstream group of neurons: glutamate is excitatory, GABA (gamma-aminobutyric acid) is inhibitory and dopamine is a modulatory neurotransmitter. The process of action selection is expressed by the direct, indirect, and hyperdirect pathways of the BG, modulated by dopamine. The premotor cortex (PMC) selects a response via direct projections from the input gated by BG through the thalamus

The cortico-basal ganglia-thalamo-cortical loop architecture in Fig. 2 is synthesized by a spiking neural network model (Fig. 3). The network learns to select one of four responses to different input stimuli. Direct and indirect pathways enable to learn the conditions that are appropriate for gating as well as those for suppressing. Differing sub-types of neurons are organized within separate layers; within layers, parallel reverberating activation loops for each action are represented in a column of units; such sub-loops independently modulate each response, allowing selective facilitation of one response with concurrent suppression of the others. Projections from the SNc to the striatum incorporate the modulatory effects of DA. Simulated phasic bursts and dips in SNc firing ensue from correct and incorrect responses, respectively, and drive learning by preferentially activating the direct pathway after a correct response and the indirect pathway after an incorrect response. The network is trained on a simulated version of a long-term motor skill training task.

The number of neurons in each sub-population and the number of connections were chosen according to Frank [3, 20], and are provided in Table 1.

**Table 1:** Network and connection parameters

Parameter	Value	Description
$N_{\text {network}}$	131	Network size
$N^{\text {striatum-Go}}$	36	Size of striatum-Go population
$N^{\text {striatum-NoGo}}$	36	Size of striatum-NoGo population
$N^{\text {GPi}}$	8	Size of GPi population
$N^{\text {GPe}}$	4	Size of GPe population
$N^{\text {STN}}$	9	Size of STN population
$N^{\text {SNc}}$	4	Size of SNc population
$N^{\text {input}}$	18	Size of input population
$N^{\text {thalamus}}$	4	Size of thalamus population
$N^{\text {PMC}}$	8	Size of PMC population
$N^{M1}$	4	Size of M1 population
$K_{\text {striatum-Go}}^{\text {input}}$	432	Number of input connections on striatum-Go neurons
$K_{\text {striatum-NoGo}}^{\text {input}}$	432	Number of input connections on striatum-NoGo neurons
$K_{\text {striatum}}^{\text {striatum}}$	5184	Number of striatal interneurons connections
$K_{\text {striatum-Go}}^{\text {PMC}}$	288	Number of PMC connections on striatum-Go neurons
$K_{\text {striatum-NoGo}}^{\text {PMC}}$	288	Number of PMC connections on striatum-NoGo neurons
$K_{\text {striatum-Go}}^{\text {SNc}}$	144	Number of SNc connections on striatum-Go neurons
$K_{\text {striatum-NoGo}}^{\text {SNc}}$	144	Number of SNc connections on striatum-NoGo neurons
$K_{\text {GPi}}^{\text {striatum-Go}}$	288	Number of striatum-Go connections on GPi neurons
$K_{\text {GPi}}^{\text {GPe}}$	32	Number of GPe connections on GPi neurons
$K_{\text {GPi}}^{\text {STN}}$	72	Number of STN connections on GPi neurons
$K_{\text {GPe}}^{\text {striatum-NoGo}}$	144	Number of striatum-NoGo connections on GPe neurons
$K_{\text {STN}}^{\text {GPe}}$	36	Number of GPe connections on STN neurons
$K_{\text {STN}}^{PMC}$	72	Number of PMC connections on STN neurons
$K_{\text {PMC}}^{\text {input}}$	96	Number of input connections on PMC neurons
$K_{\text {PMC}}^{\text {thalamus}}$	32	Number of thalamus connections on PMC neurons
$K_{\text {thalamus}}^{\text {PMC}}$	32	Number of PMC connections on thalamus neurons
$K_{\text {thalamus}}^{\text {GPi}}$	32	Number of GPi connections on thalamus neurons
$K_{\text {M1}}^{\text {PMC}}$	32	Number of PMC connections on M1 neurons

In the following, the layers and their synaptic connections are described (see Fig. 3). We provide for each layer the description of its input/output synapses contextualized to their functionality, whereas we deal with exogenous sources in a separate subsection.

***Striatum***

Direct (Go) and indirect (NoGo) pathways start from two distinct populations of neurons in the striatum, expressing D1 and D2 receptors, respectively. In our neural network, the four leftmost columns of the striatum represent the direct pathways or Go, whereas the four rightmost columns represent the indirect pathways or NoGo. Each column is involved in the selection/inhibition of a particular response (R1–R4). The Go columns project to the corresponding columns in the GPi, whereas the NoGo columns project to the corresponding columns in the GPe. Striatal interneurons are responsible for tonic inhibitory activity in the striatum and are critical for shaping neuronal circuit activity in it, particularly for modulating the activity of medium-sized spiny neurons [32]. These interneurons have been modeled as synaptically weak inhibitory connections among striatal neurons.

***GPi***

Each column in the GPi tonically inhibits the associated column of the thalamus, which is reciprocally connected to the PMC. If Go activity is stronger than NoGo activity for a response, the corresponding column of the GPi will be suppressed, removing the tonic inhibition of the corresponding thalamus unit, and facilitating its execution in the motor cortex. The inhibitory connection between cortex–striatum–GPi–thalamus–cortex implements the direct pathway.

***GPe***

GPe columns inhibit the associated columns in GPi, so that striatal Go and NoGo activity have opposing effects on the GPi. The inhibitory connection between cortex–striatum–GPe–GPi–thalamus–cortex represents the indirect pathway.

***STN***

In our model, the excitatory projections from the STN to the GPi convey cortical excitation and represent the hyperdirect pathway, which prevents premature choices suppressing movement through the elevation of GPi activity. The indirect pathway interacts within the STN with an inhibitory connection.

***SNc***

The BG nuclei receive the reward signal from the SNc through the nigrostriatal DAergic projections, which incorporate the modulatory effects of DA. All the units of SNc excite Go columns (D1 receptors) and inhibit NoGo columns (D2 receptors).

***Cortex and thalamus***

In line with the hypothesis that the BG modulates the execution of “actions” (e.g., motor responses) being considered in different areas of the cortex, we consider a simple cortical hierarchy, consisting of a single input layer, conveying the aggregated information, or encoding states, derived from different cortical contexts (sensory, motor programs, etc.), and a two-level output hierarchy comprising PMC and M1. Two sets of synapses are plastic: (1) connections from the input cortex to the PMC (to learn category–response associations) and (2) connections from the input cortex to the striatum (to learn stimulus–response associations). The synapses of the first group are based on a Hebbian learning mechanism and those of the second group on DA-mediated reinforcement learning. The thalamocortical system models the reverberating activation from BG to the cortex without plasticity.

### External inputs

The input stimulus is modulated through a simulated distinct activation of columns in the input layer, representing the condensed information of a contextualized environment state.

Tonic levels of DA are simulated by setting the SNc units to be semi-active (0.5 spikes/ms) in the response phase. Then, if the response given by the network is correct, a phasic increase in SNc firing occurs in the feedback phase, with all SNc units set to have a high firing rate (1.0 spikes/ms), causing a burst of DA, representing the reward. For an incorrect response, a phasic dip of DA occurs, with all SNc units set to zero activation (0 spikes/ms).

Throughout the network, neuronal populations receive external excitatory synaptic input according to Poisson’s distribution, to achieve realistic baseline firing rates and simulate both noise and background activity originating from other brain structures. In particular, the mean firing rate of the Poisson generator to induce noise for PMC is 0.5 spikes/ms, for GPi 16 spikes/ms, and for GPe 4 spikes/ms.

### Neurons and synapses

All neurons in the network were realized using the LIF model with exponential function-shaped postsynaptic conductance, which has been shown to be consistent with experimental data on the parameters characterizing in vivo-like activity of cortical neurons [33–35] and being adopted in several models of the BG using spiking neurons [6, 36, 37]. The standard value of the model parameters are given in Table 2.

**Table 2:** Standard LIF Neuron parameters

Parameter	Value	Description
$C_{\text {m}}$	2.0	Membrane capacitance
$g_{\text {L}}$	0.2	Leaky conductance
$E_{\text {L}}$	$-$70.0	Leak reversal potential
$V_{\text {reset}}$	$-$70.0	Reset potential of the membrane
$V_{\text {th}}$	$-$40.0	Spike threshold
$tau_{syn\_ex}$	0.5	Rise time of excitatory synaptic conductance
$tau_{syn\_in}$	10.0	Rise time of inhibitory synaptic conductance
$E_{\text {ex}}$	0.0	Excitatory reversal potential
$E_{\text {in}}$	$-$85.0	Inhibitory reversal potential
$I_{\text {e}}$	0.0	Constant input current
$t_{\text {ref}}$	1.0	Duration of refractory period

The projections from input to the striatum and to the PMC are plastic. All remaining connections of the direct, indirect, and hyperdirect pathway and the recurrent interactions within BG are static conductance-based synapses determined to assure the fulfillment of a set of functional restrictions by a robustness analysis and are in line with those used in Frank’s models [3, 20]. The value of the synaptic connectivity parameters are given in Table 3.

**Table 3:** Synaptic parameters in healthy condition

Weight	Value (nS)	Model
$w^{\text {input}}_{\text {striatum-Go}}$	*U*(0.02, 0.12)	exc
$w^{\text {input}}_{\text {striatum-NoGo}}$	*U*(0.02, 0.12)	exc
$w^{\text {striatum}}_{\text {striatum}}$	*U*(0.01, 0.04)	inh
$w^{\text {PMC}}_{\text {striatum-Go}}$	*U*(0.4, 0.5)	exc
$w^{\text {PMC}}_{\text {striatum-NoGo}}$	1.0	exc
$w^{\text {SNc}}_{\text {striatum-Go}}$	0.15	exc
$w^{\text {SNc}}_{\text {striatum-NoGo}}$	0.3	inh
$w^{\text {striatum-Go}}_{\text {GPi}}$	1.5	inh
$w^{\text {GPe}}_{\text {GPi}}$	1.0	inh
$w^{\text {STN}}_{\text {GPi}}$	6.0	exc
$w^{\text {striatum-NoGo}}_{\text {GPe}}$	1.0	inh
$w^{\text {GPe}}_{\text {STN}}$	0.35	inh
$w^{\text {PMC}}_{\text {STN}}$	*U*(0.45, 0.85)	exc
$w^{\text {input}}_{\text {PMC}}$	*U*(0.0045, 0.055)	exc
$w^{\text {thalamus}}_{\text {PMC}}$	2.0	exc
$w^{\text {PMC}}_{\text {thalamus}}$	1.5	exc
$w^{\text {GPi}}_{\text {thalamus}}$	1.0	inh
$w^{\text {PMC}}_{\text {M1}}$	2.0	exc

### Learning rule

The projections from the cortical input layer to the BG (striatum) and to the cortex (PMC) are learnable by two different learning mechanisms adopting the same learning rule. While early in training the BG learns the correct association between input and output to ensure that the correct motor plan in PMC is consistently activated shortly after the stimulus is presented (using DA-mediated reinforcement learning), the consistent association between input and PMC activity triggers Hebbian learning between input and premotor areas, and the direct cortico-cortical connections eventually become strong enough so that the BG is no longer required to produce a response. When the response process becomes purely cortical, the skill is said to be “automatic”.

For cortico-striatal learning, we have used a reinforcement learning version of Leabra, the rule proposed by Frank [3], which is more biologically plausible than standard error backpropagation. To simulate feedback effects, the learning algorithm involves the response and feedback phases. In the response phase, the network settles into activity states based on input stimuli and network synaptic weights, ultimately choosing a response. In the feedback phase, the network resettles in the same manner, with the only difference being a change in simulated DA release (i.e., SNc activity) depending on the selected response: DAergic neurons increase from tonic to phasic (i.e., high) level of activity, whereas an incorrect response causes a decrease from tonic to zero level of activity.

In specific, the rule uses a combination of error-driven and Hebbian learning. The error-driven component computes a simple difference between a pre- and post-synaptic activation product across the two phases. For Hebbian learning, we use the same learning rule used in competitive learning. The error-driven and Hebbian learning components are combined additively at each connection to produce a net weight change.

The equation for the Hebbian weight changes adopted is

1 $$\begin{aligned} \Delta _{\text {hebb}}w_{ij} = x_i^+ y_j^+ - y_j^+ w_{ij} = y_j^+ (x_i^+ - w_{ij}), \end{aligned}$$

where *x* is the presynaptic activation, *y* is the postsynaptic activation, and $-/+$ represents the response/feedback phase, respectively. The equation for the error-driven component is

2 $$\begin{aligned} \Delta _{\text {err}}w_{ij} = (x_i^+ y_j^+) - (x_i^- y_j^-), \end{aligned}$$

which is subject to a soft-weight bounding to keep within the 0–1 range:

3 $$\begin{aligned} \Delta _{sberr}w_{ij} = [\Delta _{err}]^+(1-w_{ij}) + [\Delta _{err}]^-w_{ij}. \end{aligned}$$

The two terms are then combined additively:

4 $$ \begin{aligned} \Delta_{{w_{ij}}} = \epsilon [k_{{hebb}} (\Delta _{{hebb}}) + (1-k_{{hebb}})(\Delta _{ {sberr}})], \end{aligned}$$

where $\epsilon$ represents the learning rate parameter and $k_{{hebb}}$ is a parameter that controls the associated proportion of the two types of learning.

The equations point out that plasticity depends only on the reward value. Therefore, the mechanism is a reward value-based learning that triggers plasticity regardless of what was expected. Note that an explicit supervised training signal is never presented; striatal connection weights change depending on the difference between activity states in the response and feedback phases, which only differ due to phasic changes in DA. The striatum learns over time which responses to facilitate and which to suppress in the context of incoming sensory input. Long-term potentiation (LTP) or long-term depression (LTD) occurs if the neuronal activity is increased (or decreased) with respect to the response phase.

In addition, the PMC itself learns to favor a given response for a particular input stimulus, via Hebbian learning from the input layer (Eq. 1).

The values of the parameters regarding the learning mechanism result from neurophysiological findings and are reported in Table 4.

**Table 4:** Learning rule parameters for the connection between the input cortex (PMC) and the BG (striatum) and associated simulation parameters

Description	Value
$k_{{hebb}}$	0.01
$\epsilon _{{cor}}$	0.00001
Cortico-striatal learning rate for LTD	0.1
Cortico-striatal learning rate for LTP	0.1
Step size of simulation	2 ms
Minimum time of simulation	400 ms
Maximum time of simulation	800 ms
Time window for firing activity	60 ms
Threshold firing rate	0.25
Duration of the feedback phase	200 ms
Time window for striatal learning	60 ms
Time window for cortical learning	60 ms

In short, the BG initially learns which response to gate via phasic changes in DA ensuing from random cortical responses, and then this learning transfers to the cortex once it starts to select the correct response more often than not. This implements the idea that the BG modulates the gating of responses that are selected in the cortex.

## Experimental settings

The BG is crucial in learning the cortico-striatal association to accurately control and execute complex movements, which involve the combination of elementary motor elements in a particular order with certain timing. This mechanism strictly depends on the structural integrity and functional activity of the cortico-striatal loop. To simulate these processes in the BG, we have simulated a motor program by providing the network with a set of input stimuli and evaluated the neural activation within the network and the provided neural answers. The simulations were repeated 30 times for each group (each network starts with different initial random weights) to improve the robustness of the results. Statistical analysis was carried out through paired t-tests corrected with the Bonferroni procedure.

### Task description

**Fig. 4:**
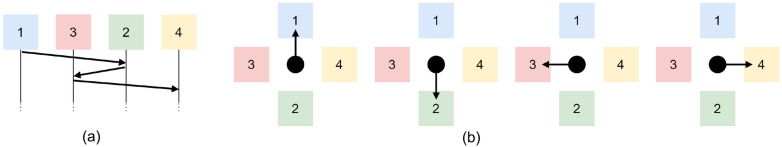
Simulated motor task. Given an input signal, the network has to learn the most appropriate motor command in response to each stimulus. The task can be schematized either as (**a**) or (**b**), and it is correctly performed when the network selects the appropriate sequence of motor commands for the whole sequence of stimuli. The sequence of stimulus–response presented to the network is that reported in the figure, from stimulus 1 to stimulus 4

The network is provided with four different input stimuli, each corresponding to an associated cortical motor response. In each trial, one of the four stimuli is presented for the whole duration of the simulation and the network has to learn the correct action to perform. Thus, the execution of the whole task includes four trials, one for each stimulus. Consequently, one epoch of the network learning process corresponds to one execution of the task, and we assume that the task has been perfectly learned once the network provides the correct actions in 10 successive executions. The sequence of stimulus–response presented to the network is that reported in Fig. 4, from stimulus 1 to stimulus 4. However, a different sequence of stimuli or a random presentation of them does not influence the network learning curve, as for each network simulation, according to the initial state of the network (in terms of initial neural weights) and background neural activity, each one of the available neural pathways becomes specialized for one of the 4 stimuli presented. As a consequence, the learning capability of the network does not depend on the order of stimuli presentation.

### Healthy and pathological conditions: simulations and performance evaluation

The simulations have been carried out for 300 epochs to dynamically assess the behavior of the networks and the steady-state level for different conditions.

To qualitatively measure the trend of the learning curve, we have defined three successive phases of the learning process as follows: (i) an early phase, going from the beginning of the simulation to the time when the average error falls below chance ($50\%$); (ii) a progressing phase, from the end of the early phase to the fulfillment of learning, defined as the time when the average error remains below $5\%$ for 10 consecutive epochs; (iii) a consolidation phase, from the end of the progressing phase and continuing for the remaining time of the simulation. It follows that the time for perfectly learning the task, here referred to as learning time, is given by the number of epochs to complete the early and progressing phases mentioned above. The response time is the time (in milliseconds) the network takes to choose an answer.

To simulate the diverse PD stages, different levels of SNc lesions have been simulated in the network. As reported in Chen et al. [38], in Parkinson’s disease the surviving neurons in SN exhibit altered firing activity, such as decreased spontaneous firing rate and reduced number of firing neurons. The firing rate reduction is achieved by setting to specific values the rate of the Poisson process driving the SNc unit, considering a range of values from 1 to 0, as a rate multiplier (with 1 corresponding to a healthy condition and 0 corresponding to absent firing rate in the neural population of SNc, respectively). We set the DA levels to 1.0 and 0.5 to tune the model into healthy and PD-symptomatic conditions respectively. In particular, DA levels ranging from 1.0 to 0.6 simulate preclinical stages (before PD diagnosis and motor symptoms), which correspond to a degree of lesion from 0% to 40%, and from 0.5 to 0.3 simulate the late stages of the disease, which correspond to 50–70% in terms of lesion.

**Fig. 5:**
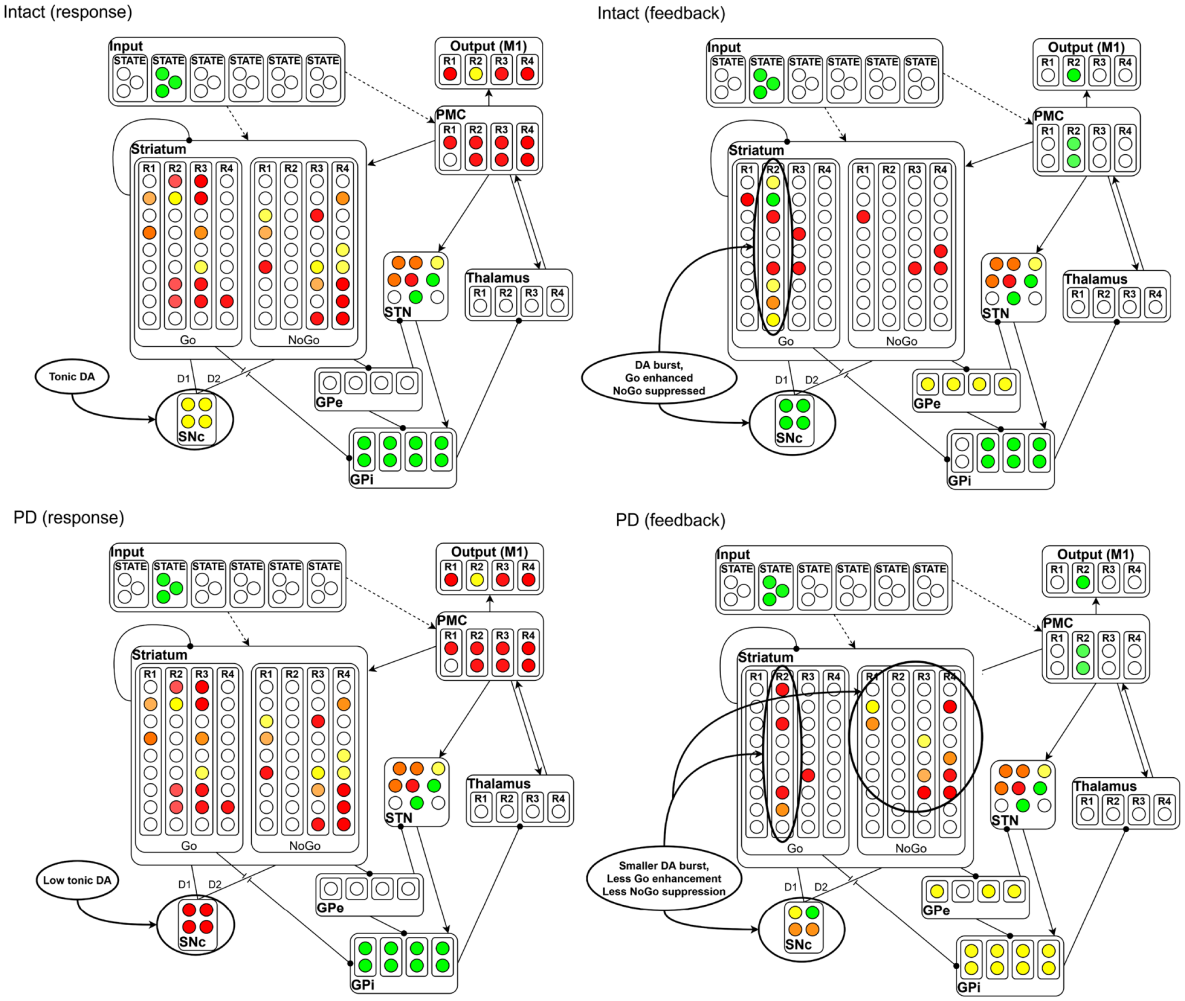
A positive feedback trial in the motor learning task, for both healthy and Parkinson networks. Red corresponds to low activation, yellow to medium, and green to high

Figure 5 shows a positive feedback trial, for both healthy and PD networks. The cue of this trial is represented by the active units in the input layer. In the healthy network, early in training, the BG has not learned to gate a response, and GPi is active, while the thalamus is not active. The PMC is weakly active due to direct connections from sensory input and SNc is tonically active. As the network guessed correctly, a phasic burst of DA firing occurs in the SNc. This has the effect of activating Go units associated with the selected response (via D1 enhancement), and suppressing NoGo units (via D2 inhibition). The enhanced Go representation in the feedback phase drives learning to gate the response. In the PD network, the reduced activity of SNc units causes the NoGo units to be more active during response selection. As the network guessed correctly, a phasic burst of DA firing occurs, but lower levels of DA lead to smaller DA bursts; therefore, DA only weakly activates more Go units and weakly inhibits NoGo activity.

Network performance is then evaluated in terms of error rate, learning time, and response time. Other parameters, calculated over 30 epochs, are percent error in the early phase, percent error in the progressing phase of the healthy condition, and percent error in the consolidation phase of the healthy condition.

## Results

DA and its phasic changes are critical for learning and executing motor responses, and a reduced dynamic range of DA is associated with PD.

With the aim of investigating how different levels of DA depletion shape BG functions, we performed a computational exploration of the role of DA deficiency in motor learning and execution within the BG using numerical simulations of the BG network. The exploration has been carried out by systematically varying the DA level and studying how gradual reductions affect the behavior with respect to the healthy condition.

### BG in healthy conditions

**Fig. 6:**
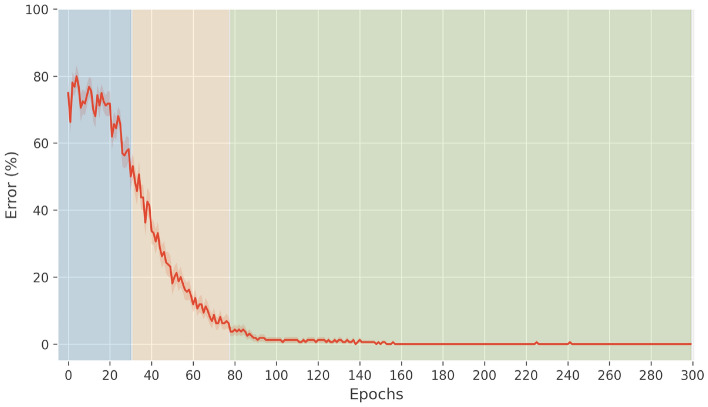
Mean learning error curve in healthy networks (averaged over 30 networks). The early phase is represented in blue, the progressing phase in yellow, and the consolidation phase in green

In healthy conditions, the activity within the striatum enables different Go and NoGo representations for various stimulus configurations, and physiological levels of dopamine allow to modulate the striatal learning. Figure 6 shows that the average learning time is 78 epochs.

#### Early phase

At the beginning of learning, random activity within the network causes the random selection of a motor response. Positive feedback (resulting from the selection of the correct motor response) leads to an increase in DA, whereas negative feedback (resulting from the selection of an incorrect motor response) leads to a decrease in DA. In turn, according to the mechanisms of synaptic plasticity within the striatum, DA increase reinforces the selection of the chosen response, whereas DA decrease prevents its selection. As long as the learning proceeds, the Go/NoGo connection weights are modeled based on the DA changes, and the network becomes able to steadily complete the task. As shown in Fig. 6, in healthy conditions the early phase lasts 30 epochs.

#### Progressing phase

During the progressing phase, the striatal weights are consistently reinforced to inhibit the GPi neurons that, in turn, disinhibit the thalamic neurons associated to the correct response. Thalamic activation results in the enhancement of PMC activation. During this phase, learning proceeds, until striatal weights become appropriate for systematically facilitating the correct responses. As shown in Fig. 6, the progressing phase lasts 50 epochs in healthy conditions.

#### Consolidation phase

During the consolidation phase, learning can be considered complete, as the network has acquired the correct associations for all the responses and the behavior is consistent in time. For each stimulus presented, the inhibition of the associated column of the GPi is substantial and robust, as well as the activity in the thalamus and PMC, because Go pathway activation is massive for the correct response and inhibited for the other responses.

### BG in pathological conditions

PD is associated with a decrease in the production of DA in the SNc, which affects the normal behavior of the BG [39]. In our model, the reduction of DA has been achieved by decreasing DAergic neurons firing rate, to reduce the dynamic range of the DA signal on the striatum. Dynamic range is critical for learning appropriate Go/NoGo representations from error feedback, as network weights are adjusted based on the differences in activity states in the two phases of network settling. Because tonic DA levels are low, PD networks have an overall propensity for NoGo learning, and Go learning is degraded because limited amounts of available DA reduce the potency of phasic bursts.

The simulation structure has been set up taking a look at clinical findings. In recent years, the Movement Disorders Society (MDS) identified three PD phases: preclinical, prodromal, and clinical PD. The preclinical phase and the prodromal phase are considered pre-diagnostic PD. The preclinical stage starts from the onset of neurodegeneration to the appearance of nonmotor symptoms; the prodromal stage represents the time interval from the onset of nonmotor symptoms to the appearance of motor symptoms (bradykinesia, rigidity, resting tremor); the clinical stage starts from the appearance of the motor symptoms to the death [40].

**Fig. 7:**
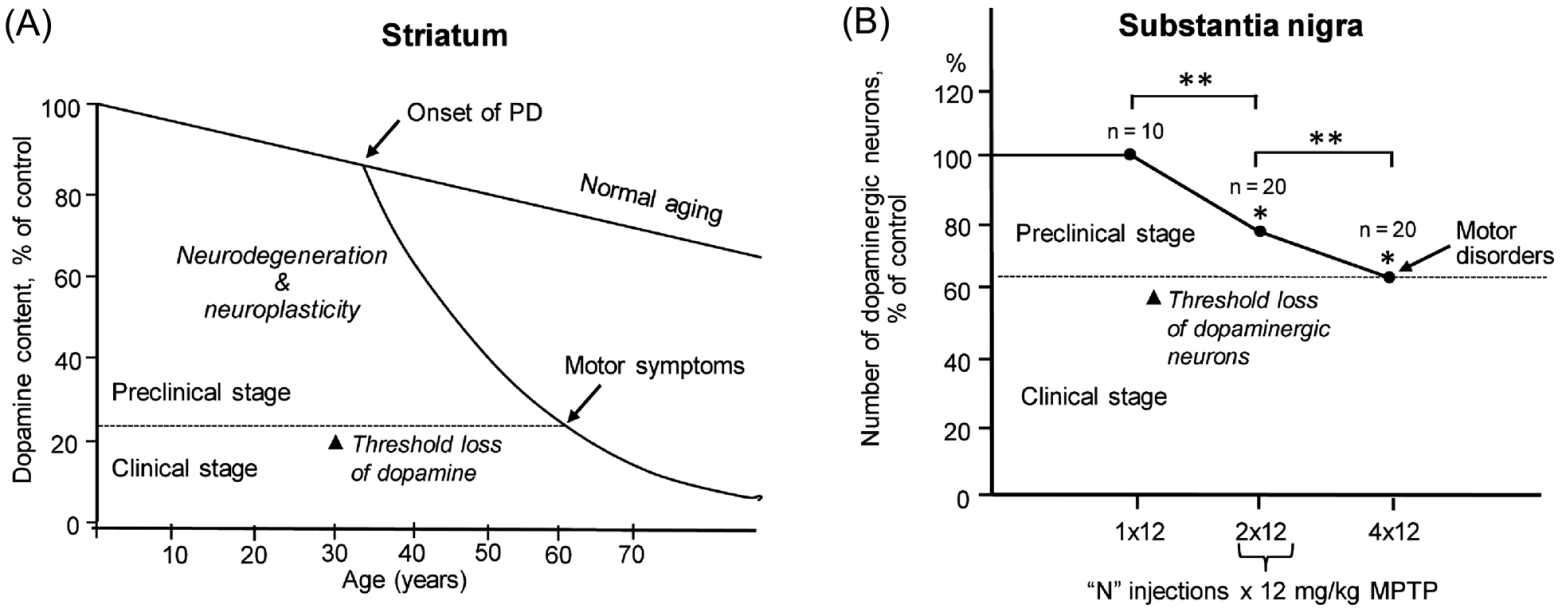
Schematic representation of the pathogenesis of PD in patients and animal models. **A** The manifestation of progressive degradation of the nigrostriatal DAergic system in patients with PD as a loss of DA in the striatum to a threshold level of dopamine, which is associated with the onset of motor disorders. **B** Progressive loss of DAergic neurons in the substantia nigra when modeling preclinical and clinical stages of PD in mice by subcutaneous injections. Reproduced from Ugrumov [40, Fig. 1A,D]

PD develops up to 30 years at the preclinical stage without manifestations of motor disorders. Neuropathological evidence suggests that motor symptoms only emerge with 60–80% depletion of DA in the striatum [41] (Fig. 7A) and 40–60% of neurons in the substantia nigra (SN) have been lost (Fig. 7B). The absence of motor symptoms until after a major degradation of the nigrostriatal dopaminergic system is due to neuroplasticity, which compensates for the failure of degenerating neurons [42].

In addition, Grosch et al. [43] claim that the loss of DAergic striatal nerve terminals at motor symptoms onset is rather difficult to determine. They provide a regression analysis to estimate the proportion of lost striatal DAergic neurons at the onset of motor symptoms comparing different studies, and it goes from 39% to 51%, in agreement with Ugrumov [40], Greffard et al. [41]. Chen et al. [9] suggest that at the time of first diagnosis of PD, only 30% or so of DAergic neurons and 50–60% of their axon terminals have been lost. In other words, at motor symptoms onset, the extent of loss on the striatum is more profound than that of DA neurons in the SN. Novel animal model studies (MPTP-treated mice) suggest the appearance of motor disorders with a 70–80% depletion of striatal DA and treated animals are strikingly similar in phenotype to PD patients [42, 44].

Currently, PD is diagnosed after the appearance of motor symptoms, generally many years after the onset of the neurodegeneration, when the patient is already at the clinical stage, reducing the efficacy of the pharmacological therapy. Therefore, a priority in PD research is to study the early stages of the disease, to diagnose the disease at preclinical or prodromal PD state, as it has many important implications. First, understanding the timing and sequence of pathologic changes could provide important clues as to the etiology and pathogenesis of the disease. Second, a pathophysiological understanding of the pre-diagnostic PD is essential for the development of new therapeutic strategies. And, finally, being able to determine that a person has PD earlier than is currently possible would permit the introduction of a putative disease-modifying therapy at a time when it could have more profound and long-lasting effects.

In this framework, thus, we want to explore the progressive loss of DA and observe emerging behaviors. Our model allows to investigate to some extent the neurobiologically plausible alterations in the learning process of motor behaviors, acting on DA content in the striatum through manipulation of the firing rate of the neurons in SNc. Among the stages of the disease, we are particularly interested in analyzing the behavior of the model in preclinical PD conditions. For this purpose, we consider the degree of lesion of 20%, 30%, and 40% as a simulation of the preclinical stage, while 50%, and 70% for simulating the clinical stage of the disease, as mentioned in Sect. 4.2. The results reported below are obtained with the lesion inflicted at the beginning of training.

#### Preclinical stage

**Fig. 8:**
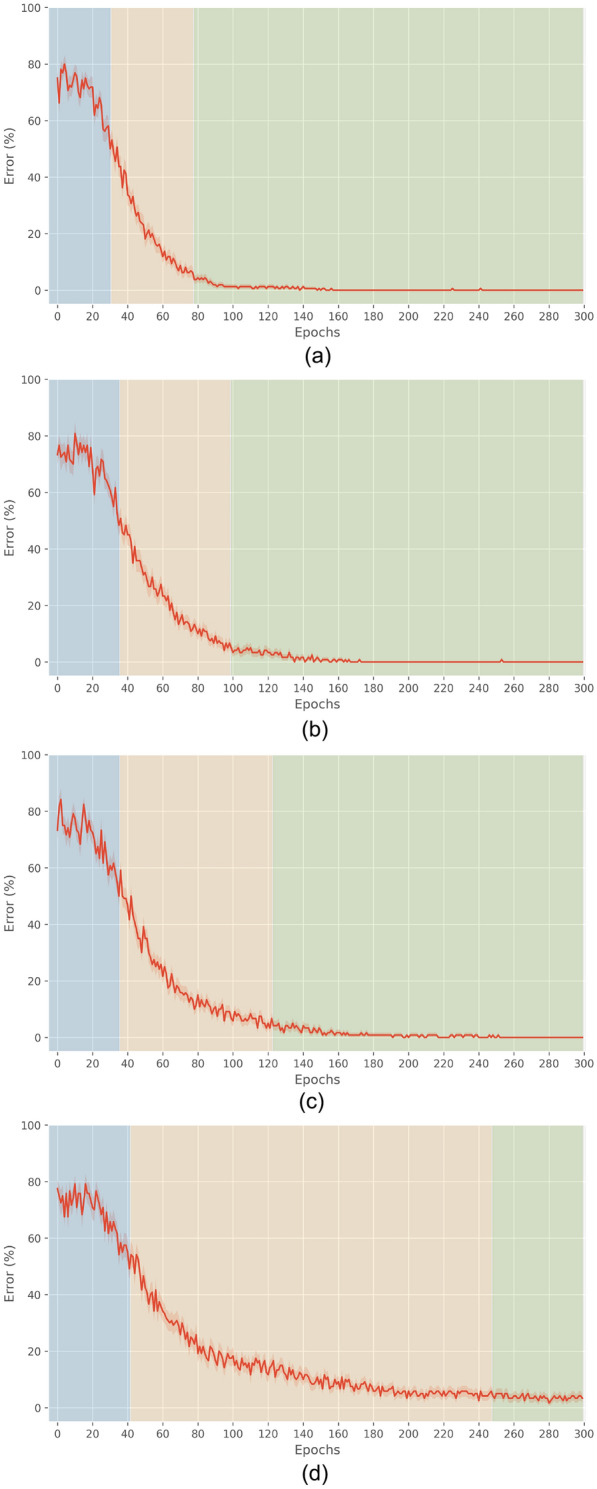
Mean learning curves (averaged over 30 networks), from healthy to early PD. Regarding the degree of lesion: **a** 0%, healthy (**b**) 20%, preclinical PD (**c**) 30%, early prodromal PD (**d**) 40%, prodromal PD

Figure 8a–d shows the learning curve in preclinical PD conditions. To facilitate the comparison, Fig. 8a shows the learning curve in healthy networks of Fig. 6. They show that the duration of the early phase (blue area) is unaffected by the extent of the lesion, while the duration of the progressing phase (yellow area) grows with it. Supporting our meta-analysis of the simulations, as we relate the range 20%, 30%, and 40% to the prodromal and preclinical stages, learning is always completed.

#### Clinical stage

**Fig. 9:**
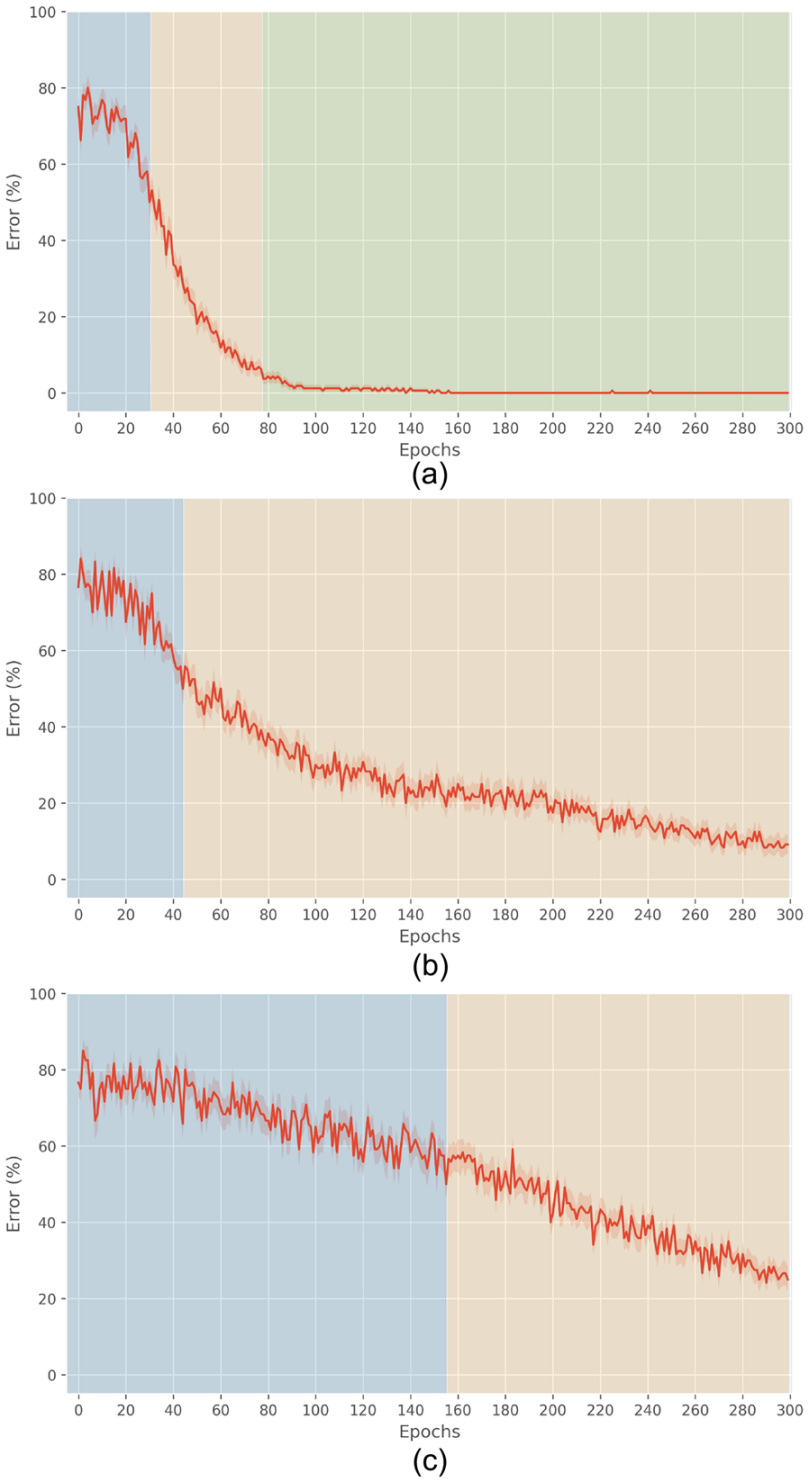
Mean learning curves (averaged over 30 networks), for healthy and late PD. Regarding the degree of lesion: **a** 0%, healthy, **b** 50%, clinical PD, **c** 70%, advanced PD

Figure 9b, c shows the learning curve in the clinical conditions, in comparison with the healthy one shown in Fig. 9a. The plots show that the knee of the curve becomes negligible with a DA depletion level of 50%, disappears with a level of 70%, and that in both cases the networks do not complete the learning. This is consistent with the observations that under those conditions the damage produces evident motor misbehavior. In line with the prediction of Frank [3, 20], the model suggests that persistent heavy DA depletion affects the steady state of the BG networks, which were impaired, and also results in stronger activity along the indirect pathway. The progressing phase (yellow area) shows an exceedingly enlarged duration with the largest extent of the lesion.

#### Comparison

**Fig. 10:**
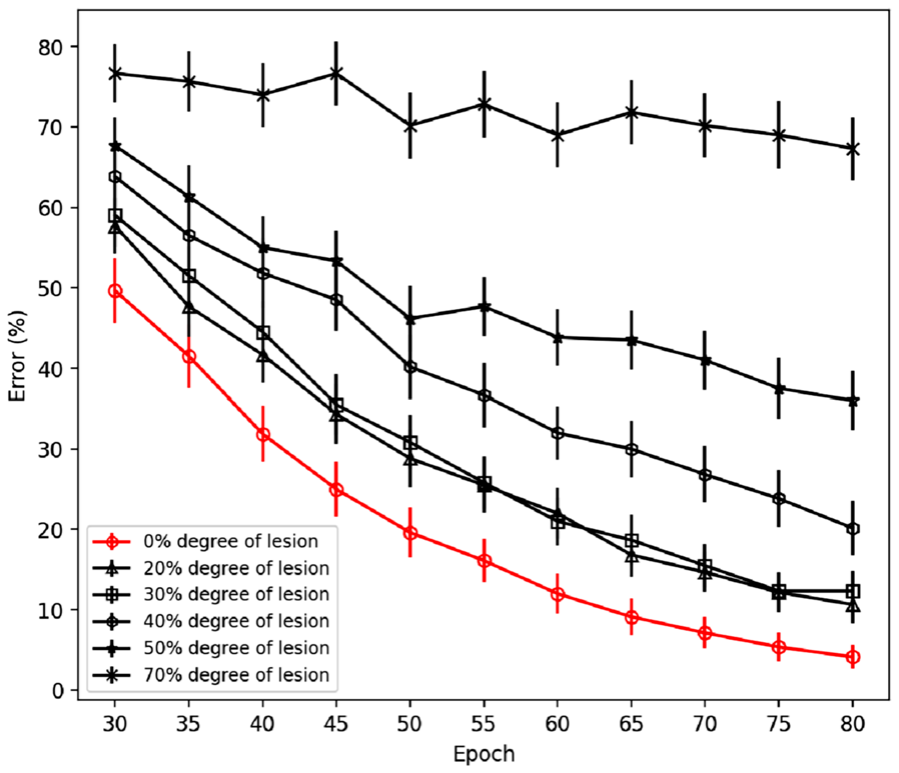
Learning curves in different conditions (intact network and different PD stages). PD networks are impaired at learning the task, due to impoverished phasic changes in DA in response to feedback. Impairment occurs also for less damaged networks

**Fig. 11:**
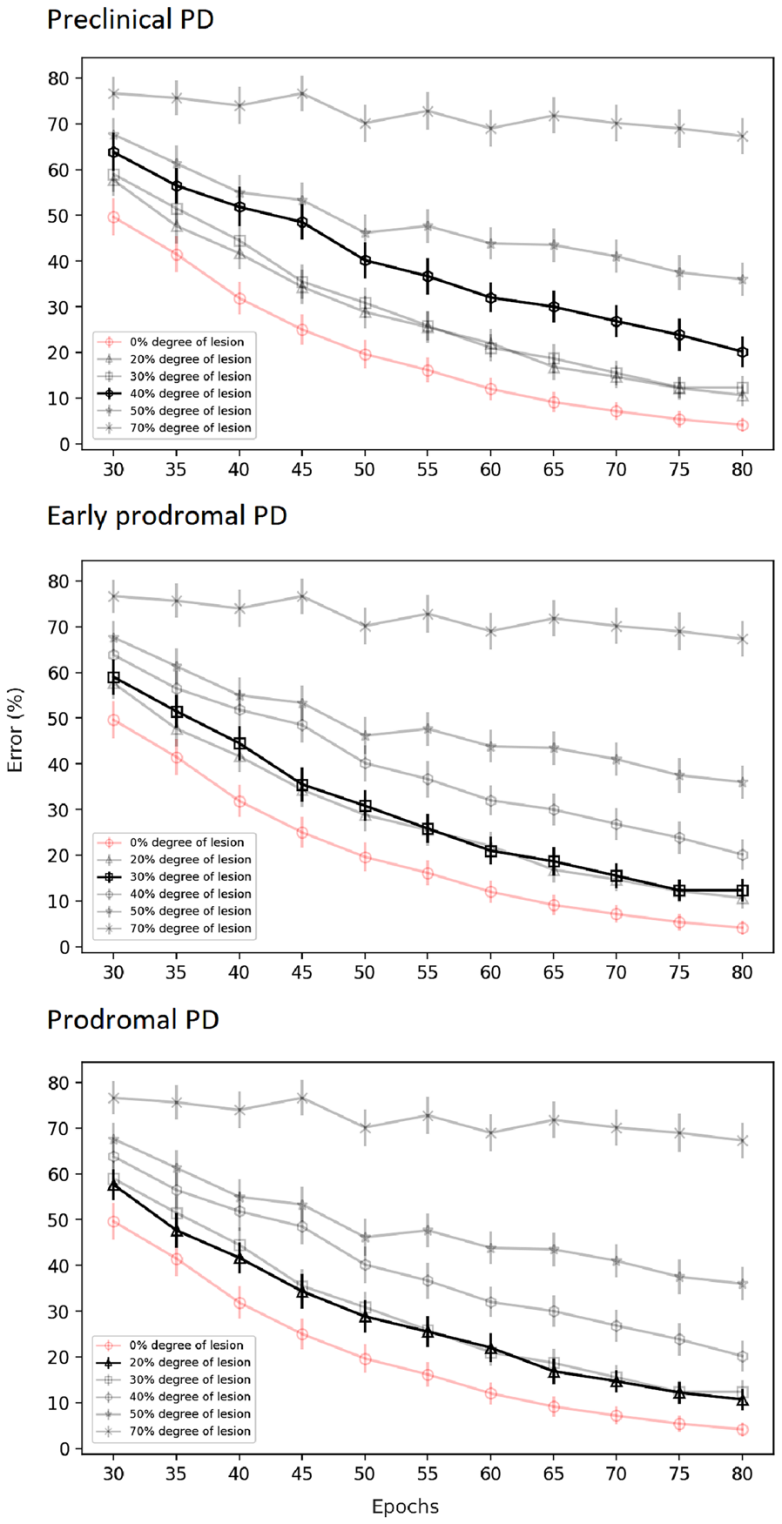
Average learning curves emphasized for simulated preclinical PD, early prodromal PD and prodromal PD

To investigate to which extent the different stages of the disease affect the performance, we focused on the behavior of networks in the time frame corresponding to the total duration of the progressing stage in healthy condition, i.e., from the 31st to the 80th epoch of the simulation, as reported in Fig. 10. As the figure shows, the healthy networks successfully learned to perform the task, i.e., achieved an error smaller than 5%. In contrast, and within the same lapse of time, PD networks were impaired, with an error ranging between 13% and 72%, depending on the extent of the lesion (Fig. 10). The difference in the performance is statistically significant even in the case of preclinical and early prodromal PD networks (*P* <.0001 for all conditions) (Figs. 10, 11).

**Fig. 12:**
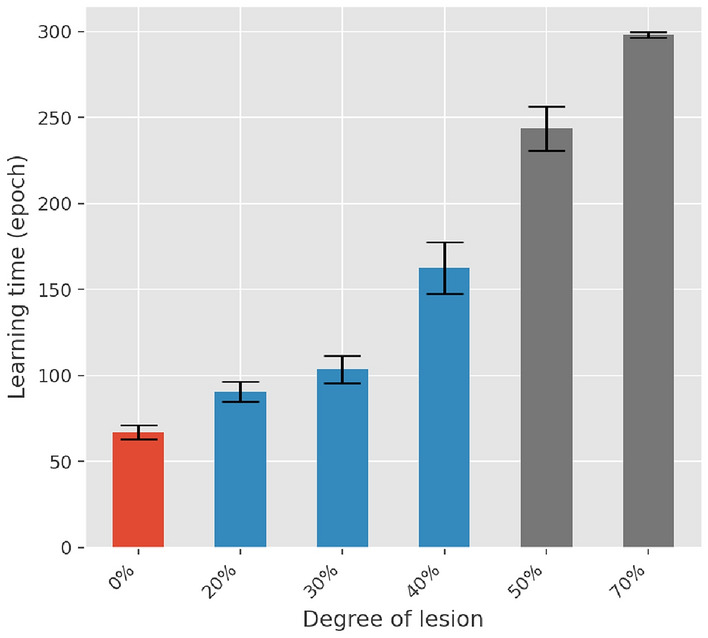
Learning times for levels of lesion ranging from 0% to 70%

**Fig. 13:**
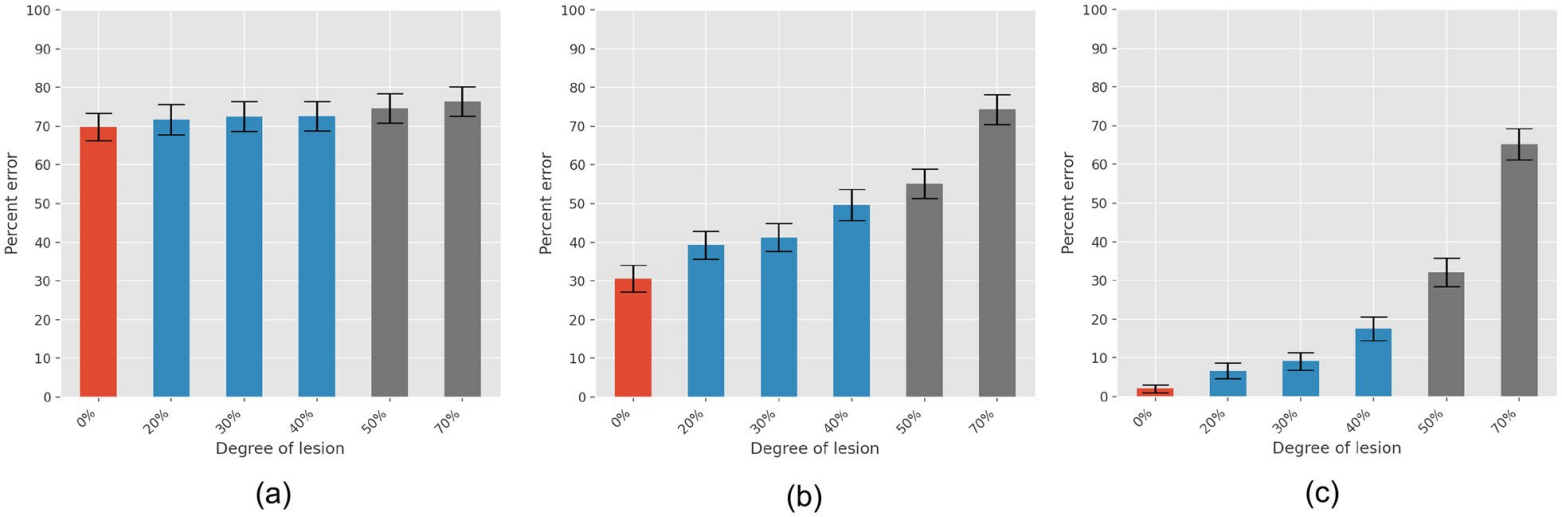
Error rates in action selection for levels of lesion ranging from 0% to 70%: **a** early phase, **b** progressing phase, **c** consolidation phase

To consider to what extent the lesions impair the learning dynamic, we compared the behavior of the networks in terms of the total learning time, and the performance of the networks across the three different phases of the learning. For this last purpose, we consider three different time intervals, each lasting 30 epochs of the simulation, but starting at the beginning of the early, the progressing, and the consolidation phase of the healthy condition, respectively. Figure 12 reports the results of such a comparison in terms of the total learning time, while Fig. 13 reports the error rates in the early, progressing, and consolidation intervals. We observed a significant difference in the learning times as a result of the DA reduction (*P* <.001 for all conditions). The same conclusion applied to the difference in both the progressing and the consolidation phases (*P* <.0001 for all conditions). In contrast, no statistically significant difference was observed in the performance during the early phase (*P* >.1 for all conditions).

**Fig. 14:**
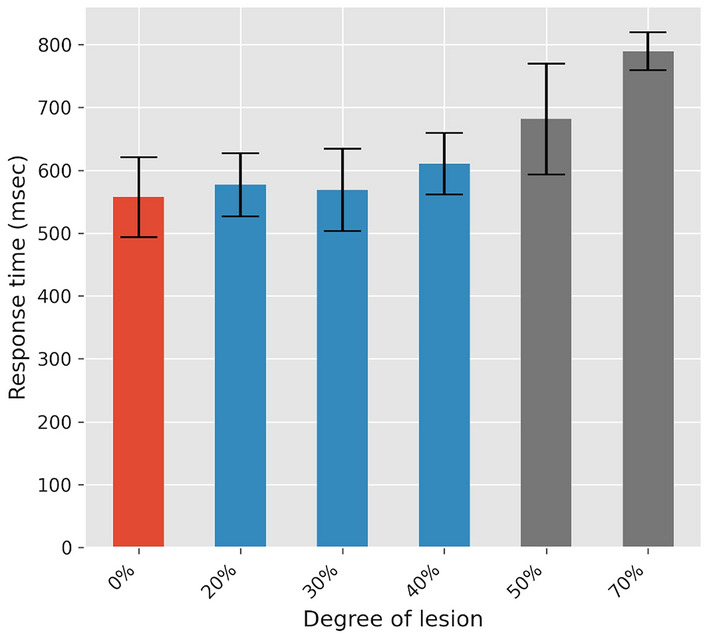
Mean response time for levels of lesion ranging from 0% to 70%

One way to assess the symptoms of PD related to motor control is the slowness of movement (bradykinesia), which we evaluated in the model with the response time. Results are illustrated in Fig. 14. We found no significant difference between healthy and preclinical PD networks (*P* >.1), while the difference is significant in the case of the clinical PD networks (*P* <.001).

The most interesting results of the experiments were revealed by the statistical analysis, which showed that there is a statistically significant difference between the healthy and the preclinical PD networks, even the ones with the smallest lesions, except in the case of the response time.

To investigate the internal dynamic of the network with the lesion of firing rate, we analyzed the activations of the modules of the network within each simulation. We examined the evolution of the striatal activity as the network is incrementally damaged. The activation rate of the columns in the direct and indirect pathway shows a progressive not significant degradation as the damage inflicted increases. As expected, in damaged networks we observed that Go learning is degraded, because reduced DA content results in reduced activation of Go neurons, and NoGo learning has proceeded, leading to the disinhibition of GPe neurons. More interestingly, the neurons in STN result overexcited, leading to the disinhibition of GPi and not to select the response. The activity of STN is in the form of occasional bursts, which cause the impairment of learning.

Eventually, further experiments were aimed at evaluating the effect of DA lesions during and after learning. We observed that damaging the networks during learning slightly deteriorated network performance with respect to the healthy condition. Instead, by damaging the networks after learning, as the BG response is dominated by strengthened cortico-striatal synapses, we observed that there was no significant difference with the healthy condition. This is consistent with the findings that PD patients are able to continue performing already learned motor tasks, although not smoothly.

## Discussion

It is well established that dopamine in the BG plays a key role in several cognitive and motor functions, by facilitating the normal BG activity during motor control and acting as a reward prediction error during motor skill acquisition [45]. The progressive degeneration of dopaminergic neurons in the pars compacta of substantia nigra and, consequently, the dysfunction of the BG, is the major neuropathological change characterizing PD.

PD affects millions of people worldwide and its treatment and evolution are closely tied to the time of diagnosis. In terms of clinical manifestation, PD causes a variety of motor and non-motor symptoms and signs, such as bradykinesia, muscular rigidity, shuffled gain, resting tremors, apathy, anhedonia, depression, and anxiety. Non-motor symptoms can appear decades before the cardinal motor features of PD start to appear, at which point the disease is eventually diagnosed [46]. As motor control symptoms appear after the degeneration of most dopaminergic neurons [40], while non-motor symptoms appearing during the prodromal stage could be associated with other neurological diseases, it is important to develop novel strategies for the early diagnosis of the disease.

With the aim of addressing this issue, we have investigated whether other motor abilities, such as motor learning, which are impaired by the disease, could be analyzed to achieve an early diagnosis, at least in the prodromal stage. We have performed this investigation through a computational approach, by developing a neural network mechanistic model of the BG, which incorporates known biological constraints and simulated different degrees of dopaminergic neuron degeneration. The BG model incorporates the three main pathways subserving the BG activity: direct, indirect, and hyperdirect pathways.

The behavior of the network in learning a simple novel motor task has been analyzed, and the model response, in terms of neural activation, was recorded and analyzed. The robustness of the network to small perturbations of the parameters was also verified, as we observed no significant difference in network behavior for realistic changes in the neural parameters.

The analysis of the model behavior in both healthy and pathological conditions provides a theoretical basis for motor learning dysfunctions associated with the alterations of the dopaminergic system in the BG. Consistent with other experimental and model-based studies, the proposed model confirms the key role of dopamine and its phasic changes as a rewarding signal in motor skill acquisition and learning of stimulus–response association. Furthermore, the analysis of the neural activation within the network, as well as of the response error rate during learning, and of the response time of the BG model in pathological conditions, provides a working hypothesis that can be tested experimentally and behaviorally.

We have simulated different stages of the disease by reducing the amount of DA through the manipulation of the firing rate in SNc and, thus, of its modulatory effects on Go and NoGo representations. According to the learning behavior shown by the model, even a small lesion in the DA system ($20\%$) impairs the ability of the network to learn a novel motor task, as learning is slowed down and the dynamic properties observed in the learning curves significantly differ from those observed for the healthy networks. This behavior suggests that learning symptoms arise already at the very early stage of the disease. As expected, higher levels of DA degeneration cause more evident learning deficits.

To investigate whether the model fits the experimental data regarding DA depletion and motor symptoms [40], we have also evaluated the response time of the model in both healthy and pathological conditions. Obtained results show that motor symptoms, in terms of response time, arise after $50\%$ of DA depletion, as we observed a significant increase in the time interval needed for selecting a motor response compared to the physiological conditions.

Taken together, these results suggest that learning abilities are affected by the disease before the motor ones, and therefore provide an important insight into developing novel diagnostic procedures, aimed at achieving early detection of the disease. Indeed, diagnostic tests aimed at evaluating motor learning abilities besides those related to motor control could be useful for achieving an early diagnosis of PD. This, in turn, would be very beneficial in adopting specific and more effective therapies at the earliest stages of the disease.

In summary, the results obtained provide working computational feedback for the hypothesis of deteriorated behavior in motor learning tasks since preclinical PD condition, which can be further tested experimentally and behaviorally at different scales and more granularity. Also, findings give an indication that subjects should be tested on the execution of novel and relatively complex motor tasks, and that parameters of the dynamics producing the movement in the learning process should be evaluated to differentiate between healthy and non-healthy undiagnosed subjects not only for an early diagnosis, but also for assessing the stage of the disease and the definition of novel rehabilitation protocols and other treatments.

## Conclusion

The computational study of neurodegeneration in the BG revealed that motor skill learning is affected by DA depletion and even a small loss of DA, which may be associated with the early stage of PD, interestingly alters the process. The model behavior in pathological conditions, with different levels of simulated DA degeneration, shows that motor learning impairments in PD arise before the onset of motor control deficits, suggesting that there may exist abnormalities of the motor learning process, due to alterations in the dynamics of the neuronal populations included in the cortex–basal ganglia–thalamus–cortex network, which do not yet involve the presence of symptoms that normally lead to the clinical diagnosis. This work provides the direction for further experimental studies investigating learning profiles, and biological and behavioral parameters in learning new motor skills. The characterization of those parameters may represent a significant stepping stone toward the research of the issue.

## Data Availability

Data sharing is not applicable to this article as no datasets were generated or analysed during the current study.

## Code availability

The code to simulate the network is available on request and free of charge for research purposes. It is written and executed in Python 3.8.10 and requires NEST 2.20.0 [47] to run.
